# Role of Robotic-Assisted Surgery in Public Health: Its Advantages and Challenges

**DOI:** 10.7759/cureus.62958

**Published:** 2024-06-23

**Authors:** Alisha Handa, Abhay Gaidhane, Sonali G Choudhari

**Affiliations:** 1 Community Medicine, Datta Meghe Institute of Higher Education and Research, Wardha, IND; 2 School of Epidemiology and Public Health, Jawaharlal Nehru Medical College, Datta Meghe Institute of Medical Sciences, Wardha, IND; 3 School of Epidemiology and Public Health, Community Medicine, Jawaharlal Nehru Medical College, Datta Meghe Institute of Medical Sciences, Wardha, IND

**Keywords:** robotic-assisted surgery, robotic-assisted surgery advantages, challenges of robotic-assisted surgery, evolution of ras, role of public health in ras

## Abstract

The modern hospital setting is closely related to engineering and technology. In a hospital, modern equipment is abundant in every department, including the operating room, intensive care unit, and laboratories. Thus, the quality of treatment provided in hospitals and technology advancements are closely tied. Robotic systems are used to support and improve the accuracy and agility of human surgeons during medical procedures. This surgical approach is commonly referred to as robotic surgery or robotic-assisted surgery (RAS). These systems are not entirely autonomous; they are managed by skilled surgeons who carry out procedures with improved accuracy and minimized invasiveness using a console and specialized instruments. Because RAS offers increased surgical precision, less discomfort after surgery, shorter hospital stays, and faster recovery time, all of which improve patient outcomes and lessen the strain on healthcare resources, it plays a critical role in public health. Its minimally invasive technique benefits patients and the healthcare system by lowering problems, reducing the requirement for blood transfusions, and reducing the danger of infections related to medical care. Furthermore, the possibility of remote surgery via robotic systems can increase access to specialized care, reducing regional differences and advancing fairness in public health. In this review article, we will be covering how RAS has its role in public health.

## Introduction and background

Although robots have been around for a while, they are still relatively new in the medical field. The field gained popularity as a means of minimally invasive surgery in the 1980s. Even though laparoscopy was already widely used, its capabilities were somewhat restricted compared to the then-believed promise of robotic surgery. The NASA Ames Research Centre also began developing the idea of telepresence in surgery simultaneously. In the 1990s, Stanford joined them, creating a highly developed telemanipulator as the foundation for ensuing systems. The FDA authorized AESOP (Computer Motion, Inc., Goleta, CA) in 1994 [1]. The first direct interventional support by a robotically assisted surgical system (RASS) on a human patient occurred in 1985: A PUMA-200 industrial robot positioned and locked a biopsy channel during a computer tomography (CT)-guided brain biopsy in neurosurgery [2]. For nearly three decades, the robotic surgery market has seen enormous expansion, mainly in terms of innovation and advancement in medical equipment. Improved surgical results, precise procedure execution, and quick patient recovery following surgery are some of these technologies' main benefits.

A minimally invasive surgical system called the da Vinci Surgical System debuted in 1999 [3]. By the end of 2017, there were a total of 8,77,000 surgical procedures (approximately) performed by the da Vinci robotic surgical system with the help of 4,409 surgical systems installed worldwide, compared with 7,53,000 in 2016 and 6,52,000 procedures done in 2015, respectively [4]; until today, more than seven million procedures have been performed utilizing RASSs [2]. The American Computer Motion's AESOP® and ZEUS robotic surgical systems were the first to be used in general surgery [5]. Following protracted legal proceedings, American Computer Motion merged with its primary rival, Intuitive Surgical, which had been established eight years earlier in 2003 [5]. The corporation registered over 7000 patents, which was the primary impediment to competitors' development and allowed them to create multiple generations of master-slave multi-arm robots to safeguard their products [6]. The earliest registered patents gradually expire after 20 years, allowing rival products to be developed. Due to the products' purported technical advantages over laparoscopy, the Intuitive Surgical firm was able to enjoy a 20-year monopoly, which gave them a significant competitive advantage of 3D imaging, magnification, dexterity, tremor filtration, motion scaling, and a short learning curve over laparoscopy [7]. By the start of 2023, over 11 million robotic procedures had been carried out globally using Intuitive Surgical Da Vinci robots, with over 7,500 systems in use [8].

India received its first urologic robotic installation in 2006 at the All India Institute of Medical Sciences, New Delhi, following the US FDA's 2000 approval of the da Vinci system [9]. India witnessed an unparalleled boom in robotic surgery throughout the subsequent ten years. As of July 2019, our nation had 66 centers and 71 robotic installations, housing over 500 skilled surgeons. Over 12,800 surgeries have been carried out in the past 12 years with robotic help. The numbers should rise as more robotic surgeons receive training and other surgical specialties use this platform more often. The pattern indicates that robotic surgery has been and will continue to grow rapidly and significantly in India [10]. In India, private hospitals are the central locations for robotic-assisted surgery (RAS); however, many government institutions have also set up robotic surgical platforms. The cost-effectiveness of robotic surgery has been vigorously debated in developing India [10,11]. Since surgery is a relatively new area, surgeon training is crucial. Nonetheless, most resident training programs in India need a standardized curriculum for teaching robotic surgery [12]. Like other surgical methods, mastery of robotic surgery can be attained by surpassing the learning curve, typically necessitating the surgeon to execute a certain quantity of single procedures [13].

## Review

Methodology

**Figure 1:**
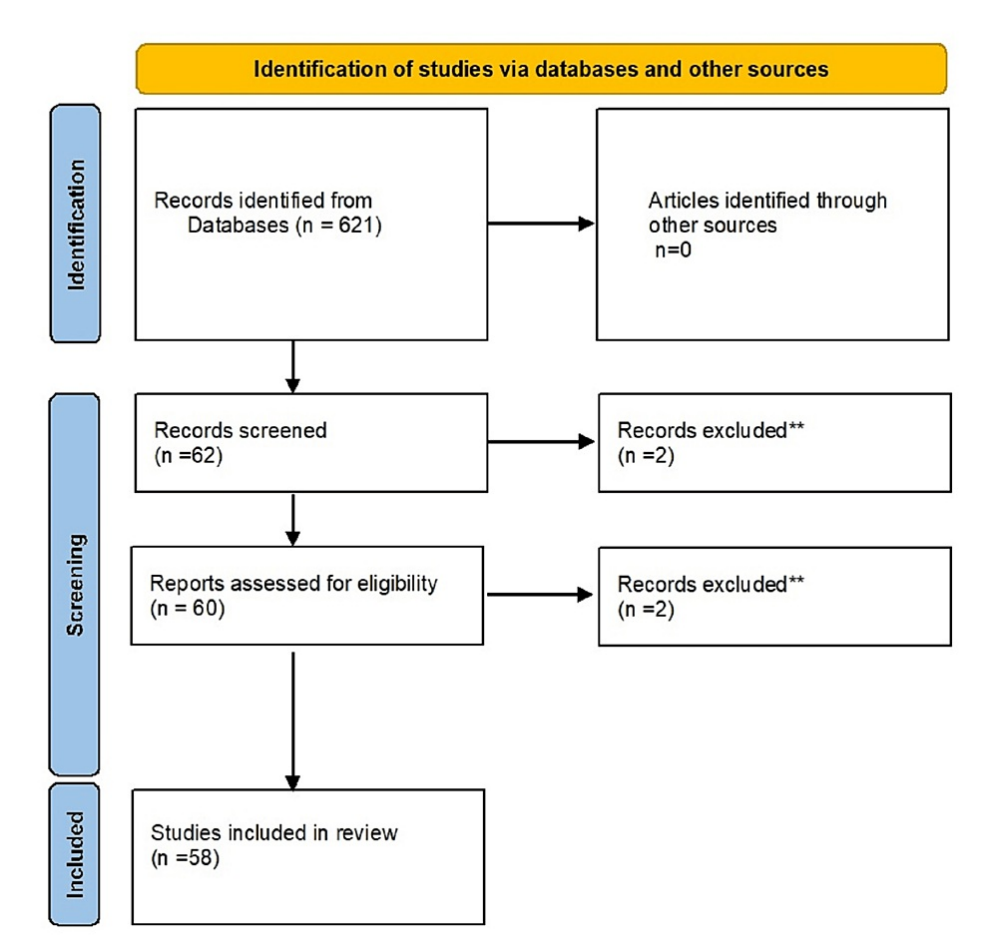
Eligibility criteria of the literature search process

The eligibility criteria for this review included all articles, studies, and documents that discussed implementing the role of RAS in public health, its operationalization, and challenges in India. For the literature search, we used electronic PubMed, Google Scholar, and Web of Science databases for relevant results. These were combined with "AND" to obtain desired results. The search was limited to publications for 10 years, from 2003 to 2023. We obtained 621 articles from the search engines using search terms like "robotic-assisted surgery” in “public health" and "challenges of robotic-assisted surgery” and their synonyms. After filtering the results by full free-text article availability and articles from 2003 to 2023, we obtained 62 articles. After screening the title and abstract, 60 articles were selected. Finally, after reading the full-text articles available, 58 articles were used for this article. Only English-language literature was included in the search parameters. The PRISMA flowchart for the methodology has been demonstrated, as shown in Figure 1.

A brief overview of the evolution of RAS is depicted in Table 1.

**Table 1:** Brief overview of the evolution of robotic-assisted surgery PUMA: Programmable Universal Machine for Assembly, FDA: Food and Drug Administration, AI: artificial intelligence Table credits: Alisha Handa

Year	Milestone
1985	First robotic-assisted surgical system PUMA 560 was used in a neurosurgical biopsy - an initial exploration of robotic capabilities in surgery.
1990s	Early research and development in robotic-assisted surgery. Focus on enhancing precision and control.
2000	Da Vinci Surgical System receives F.D.A. approval. Introduced for urological procedures. Marked the beginning of a new era in robotic surgery.
2003	Expansion of Da Vinci applications to gynecology. Widening the range of procedures amenable to robotic assistance.
2008	Introduction of single-site surgery with Da Vinci. Enabled surgery through a single incision. Reduced scarring and improved cosmetic outcomes.
2010s	Growing adoption in various surgical specialties. Increased use in urology, gynecology, and general surgery.
2014	FDA approval for Senhance surgical system. Designed for minimally invasive surgery. Added to options available to surgeons and hospitals.
2018	Continued advancements in robotic technology Improved visualization, better ergonomics, and enhanced agility. Surgeons gain greater control and precision.
2020	Increased adoption of robotic systems worldwide. Expanding applications in colorectal surgery, thoracic surgery, and more enhanced training programs for surgeons using robotic platforms.
2023	Ongoing research and development for enhanced robotic-assisted technologies Integration of AI for smarter surgical assistance. Emphasis on improving accessibility in remote and underserved areas.

Table 2 is a comprehensive table summarizing the important articles' key findings. 

**Table 2:** Summary of some important articles selected on the role of robotic-assisted surgeries in public health, its advantages, and challenges.

Author	Title	Key findings
Shah et al., 2014 [1]	The History of Robotics in Surgical Specialties	Tele-surgery, in which the physician performs the procedure while not being present in the same room as the patient, is another potential development area for robotic surgery.
Klodmann et al., 2021 [2]	An Introduction to Robotically Assisted Surgical Systems: Current Developments and Focus Areas of Research	At present, proven robotic platforms are being used more and more in research on robotically assisted surgical systems (RASSs). Miniaturized tools and semi-autonomous aid functions are designed to reduce patient trauma while maximizing the surgeon's skill.
Platis et al., 2014 [3]	Impacts of Robotic Assisted Surgery on Hospital’s Strategic Plan	The study's primary findings indicate that, while robotic surgery may not always be cost-effective, overall, and when considering all hospital-related factors, it is a worthwhile procedure to use.
Boyina et al., 2020 [4]	Robotic Surgery-Safety and Effectiveness, in Comparison with Traditional Surgery, Present Context and Recent FDA Safety Warning	Evidence from a phase III multicenter randomized trial assessing the disease-free survival state in patients following a radical hysterectomy procedure 4.5 years ago revealed that the use of robotic or minimally invasive techniques during a radical hysterectomy is linked to a higher recurrence rate compared to open approaches.
Pugin et al., 2014 [5]	History of Robotic Surgery: From AESOP^®^ and ZEUS^®^ to da Vinci^®^	It has become feasible to show the true value of robotics in minimally invasive surgery, especially in the area of bariatric surgery, as expertise with the da Vinci® robotic system in visceral surgery has grown (more than 250 significant surgeries completed).
Hughes et al., 2023 [6]	The Availability, Cost, Limitations, Learning Curve and Future of Robotic Systems in Urology and Prostate Cancer Surgery	Accessibility: The majority of them (84%), were urological operations; yet, the study does show that patient accessibility to robotic surgery facilities varies, even in a relatively small nation like England. Cost: The initial cost of procurement, as well as ongoing maintenance and consumable costs, constitute a significant barrier. Learning curves and training- The majority of the early adopters of robotic surgery in urology switched from another surgical technique (open and/or laparoscopic). As a result, trainees had less opportunities to start honing these abilities in the field because older urologists who had already finished their training had to learn how to use RAS later in their careers.
Brodie et al., 2018 [7]	The Future of Robotic Surgery	The only surgical robotic systems that are currently offered for sale that offer haptic feedback are the Senhance and MAKO RIO systems. Clinical studies contrasting the advantages of haptic feedback with no haptic feedback in these systems have not been conducted. Haptic feedback is present in most robotic systems under development, and it appears that this will set the standard for systems to come.
Marchegiani et al., 2023 [8]	New Robotic Platforms in General Surgery: What’s the Current Clinical Scenario?	In the fields of hepatobiliary, colorectal, abdominal wall, upper gastrointestinal, endocrine, and breast surgery, more and more robotic surgeries using novel robotic equipment have been reported. This review indicates that most surgical therapies are technically possible despite the low quality of the available evidence.
Coelho et al., 2010 [9]	Retropubic, Laparoscopic, and Robot-Assisted Radical Prostatectomy: A Critical Review of Outcomes Reported by High-Volume Centers	For individuals with localized prostate cancer, RRP, LRP, and RARP treatments carried out in high-volume centers are safe choices with comparable overall complication rates. However, when compared to RRP, LRP and RARP are linked to lower surgical blood loss and lower transfusion risk.
Bora et al., 2020 [10]	Robot-Assisted Surgery in India: A SWOT Analysis	Strengths: increased insurance, rising patient base, improving economy, skilled laparoscopic surgeons, training and mentorship, and a rise in the number of experienced surgeons (National Health Profile, 2018).
Udwadia et al., 2015 [11]	Robotic Surgery Is Ready for Prime Time in India: Against the Motion	The remarkable and satisfying spread of laparoscopic surgery throughout India is a notable achievement in the advancement of surgery in small towns. This can be attributed to the fervor, resourcefulness, and unwavering determination of small-town and rural Indian surgeons who have persevered through numerous challenges related to safety, innovation, and cost-effectiveness. For new technology to be useful in developing nations, it must follow the five advantages: Reasonably priced, agreeable, reachable, accessible, and suitable.
Carpenter et al., 2017 [12]	Training the Next Generation of Surgeons in Robotic Surgery	The use of robotic surgery technology has grown rapidly in many regions of the world and in many different specialties, but sadly, robotic surgeon certification and training are still in their infancy. A standardized robotics training program is long overdue and desperately required. Depending on the location and specialization of the trainee, there might be significant differences in the quality of robotic training due to the absence of a standardized training program.
Darlington et al., 2022 [13]	A Cross-Sectional Study of Resident Training in Robotic Surgery in India	The majority of residents view the introduction of robotic surgery into surgical residency programs as a danger to their training in conventional surgical techniques. This demands that resident training cases be distributed equally throughout programs across the nation and that robotic training be successfully included in residency training.
Jones et al., 2001 [14]	Surgical Aspects and Future Developments of Laparoscopy	This article examines a number of limited access techniques that are still under development, are becoming more widely acknowledged, or have been put into practice. It has been stressed that there are complete contraindications to laparoscopy.
B et al., 2002 [15]	Early Experience with Telemanipulative Robot-Assisted Laparoscopic Cholecystectomy Using da Vinci	From the first industrial robot used for stereotactic biopsies to the development of robotic guidance systems that allowed solo endoscopic surgery to the use of robotic devices for telemanipulative surgery with master-servant computer-enhanced robotic devices, the history of robotic devices is remarkable.
Fuchs et al., 2002 [16]	Minimally Invasive Surgery	Over the past ten years, all surgical specialties have seen a change in method due to minimally invasive surgery. This trend has prompted surgeons to reconsider standard practices with relation to perioperative factors like pain management, in addition to replacing conventional procedures with less invasive ones. Nevertheless, since the advent of this new technique, two significant disadvantages have surfaced: first, most surgeons have a longer learning curve than during open surgery; and second, costs have increased because of the equipment needed, the use of disposable instruments, and longer operating times.
Allendorf et al., 1997 [17]	Postoperative Immune Function Varies Inversely With the Degree of Surgical Trauma in a Murine Model	The level of surgical trauma has an inverse relationship with postoperative cell-mediated immune function. Findings from the groups that underwent laparoscopy and mini-laparotomy indicate that techniques involving tiny incisions may preserve postoperative immune function.
Lanfranco et al., 2004 [18]	Robotic Surgery	Since robotic surgery is still in its early stages, its market niche is yet unclear. Nowadays, its practical applications are mainly limited to minor surgical procedures.
Bramhe et al., 2022 [19]	Robotic Surgery: A Narrative Review	The focus is on the advancements in the usage of these devices during surgical procedures and the positive outcomes they have produced for various therapies. In this instance, the bioethical debate around robotic surgery—which is still in its infancy in academic circles and medical research—becomes extremely beneficial in assisting with decision-making when robots are involved in providing care for people.
Vermandois et al., 2019 [20]	Evaluation of Surgical Site Infection in Mini-invasive Urological Surgery	One of the most frequent surgical consequences is surgical site infection (SSI), which is linked to death, longer hospital stays, higher rates of re-admission, and a worsening of health-related quality of life.
Okhawere et al., 2023 [21]	One-Year Healthcare Costs After Robotic-Assisted and Laparoscopic Partial and Radical Nephrectomy: A Cohort Study	The cost-benefit analysis of laparoscopic surgery (Lap) against partial and radical nephrectomy (PN) is not well established, despite the widespread use of robotic-assisted surgery (RAS).
Giri et al., 2012 [22]	Current Status of Robotic Surgery	The robotic approach to prostatectomy and hysterectomy enables the advantages of laparoscopic surgery—such as reduced blood loss, reduced pain after surgery, improved cosmetic results, and a quicker return to physical activity—to the open procedures. Therefore, improved outcomes in clinical trials can be attributed to the well-established advantages of laparoscopy over open surgery.
Bankar et al., 2022 [23]	Robot-Assisted Surgery in Gynecology	One of the better choices available to women undergoing myomectomy, hysterectomy, and pelvic organ prolapse surgery is robot-assisted surgery. In addition to gynecology, other specialties like neurosurgery, orthopedic surgery, colon endoscopy, benign prostate surgery, urology, general surgery, respiratory surgery, and cardiac surgery also include robotic surgery. Robotic surgery can lower the danger of infection while improving and correcting a number of developing issues.
Upasani et al., 2023 [24]	Robot-Assisted Reconstructive Surgery of Lower Urinary Tract in Children: A Narrative Review on Technical Aspects and Current Literature	It is safe and practical to repair the lower urinary system in youngsters using robotics. Better access is provided with a robotic method, particularly in the small area inside the pelvis. It enables an earlier recovery and discharge by lowering blood loss and post-operative discomfort. Extended monitoring along with growing experience may confirm these preliminary findings.
Alaraj et al., 2011 [25]	Virtual Reality Training in Neurosurgery: Review of Current Status and Future Applications	The field of neurosurgery is beginning to use fully immersive technology. Detailed virtual reality neurosurgery modules will soon become a crucial component of the neurosurgeon training program.
Sereno et al., 2007 [26]	Telementoring for Minimally Invasive Surgical Training by Wireless Robot	In-person mentoring and hands-on training sessions are great teaching methods for laparoscopic surgery; nevertheless, financial, scheduling and geographical limitations make it impractical for specialized teachers to be present all the time. A wireless videoconferencing mobile robot used for remote robotic telementoring may be a substitute for in-person instruction.
Lam et al., 2021 [27]	Uptake and Accessibility of Surgical Robotics in England	Case volumes and nationwide accessibility to robotic treatments are inconsistent and do not provide excellent value for the National Health Service (NHS). A national registry for robotic surgery is necessary to evaluate the availability of this technology on a dynamic basis and has the potential to enhance the quality of robotic surgery.
McDonald et al., 2014 [28]	Physician Pain and Discomfort During Minimally Invasive Gynecologic Cancer Surgery	An increasing number of gynecologic oncologists describe physical issues associated with MIS. There seems to be a correlation between female sex and robotic surgery and physical discomfort. We must endeavor to enhance the ergonomics of MIS for surgeons in addition to our goal of using it to improve patient outcomes and lower patient morbidity.
Coughlin et al., 2018 [29]	Robot-Assisted Laparoscopic Prostatectomy Versus Open Radical Retropubic Prostatectomy: 24-Month Outcomes From a Randomised Controlled Study	The lack of standardization in postoperative management across the two trial groups and the utilization of additional cancer treatments warrant caution when interpreting the oncological results of our study. It is important for patients and doctors to understand that a robotic technique has many advantages, chief among them being less invasiveness.
Bochner et al., 2014 [30]	A Randomized Trial of Robot-Assisted Laparoscopic Radical Cystectomy	When compared to open surgery, retrospective studies show that robot-assisted laparoscopic surgery has a lower risk of complications and a shorter hospital stay.
Jayne et al., 2017 [31]	*Effect of Robotic-Assisted vs Conventional Laparoscopic Surgery on Risk of Conversion to Open Laparotomy Among Patients Undergoing Resection for Rectal Cancer: The ROLARR Randomized Clinical Trial *	In contrast to traditional laparoscopic surgery, robotic-assisted laparoscopic surgery did not significantly lower the likelihood of conversion to open laparotomy among patients with rectal adenocarcinoma eligible for curative resection. These results imply that there is no benefit to robotic-assisted laparoscopic surgery in rectal cancer resection when the surgery is carried out by surgeons with different levels of robotic surgery experience.
Pandav et al., 2022 [32]	Leveraging 5G Technology for Robotic Surgery and Cancer Care	Novel therapeutic uses may emerge as a result of new technical developments. Even if there are obstacles and problems with the 5G infrastructure, compatibility, cost, and security, more research is needed to understand the advantages of incorporating the technology into practice and get over the barriers before it is widely used in clinical settings. 5G-enabled remote and tele-mentored surgeries may provide a new tool for treating patients who need robotic surgical treatment, such as those with prostate cancer.
Maurice et al., 2016 [33]	Robotic Prostatectomy Is Associated With Increased Patient Travel and Treatment Delay	Access to care may be hampered by RARP's correlation with increased patient travel and treatment delays. It is yet unknown how significant these discoveries are from a therapeutic standpoint.
Mehta et al., 2022 [34]	Embracing Robotic Surgery in Low- and Middle-Income Countries: Potential Benefits, Challenges, and Scope in the Future	Socioeconomic limitations are one of the main things preventing access. Licensing universal robotic technology has the potential to reduce installation costs by increasing product availability and competitiveness. Encouragement of HICS to pool resources and equipment, the establishment of a national cloud system supported by many countries, and the creation of subsidies to enable financial support for implementation for hospitals in more remote areas are further possible strategies to lessen the burden.
Mohan et al., 2021 [35]	Telesurgery and Robotics: An Improved and Efficient Era	Although it has numerous obstacles, telesurgery, often known as remote surgery, is a promising development in surgery. For precise and well-executed procedures, zero-latency time and advancements in haptic feedback technologies are necessary. Telesurgery should incorporate technologies such as 5G networks, IoT, and haptic robotics in order to get over these obstacles. There are still costs and legalization to consider when addressing moral and legal dilemmas. By reducing the number of surgical staff members in the operating rooms, robotic surgery can play a crucial part in the surgical procedures being conducted during the present pandemic and so reduce the risk of COVID-19 infection, which can cause severe morbidity and mortality.
Ahuja et al., 2019 [36]	The Impact of Artificial Intelligence in Medicine on the Future Role of the Physician	By evaluating the enormous volumes of diverse data that patients and healthcare facilities continuously capture, artificial intelligence will help meet the demands of the medical field in the future. AI is probably going to help and enhance doctors by eliminating the repetitive aspects of their job, which should allow them to spend more valuable time with their patients and provide a better human touch. Medical personnel must understand the foundations of AI technology and how AI-based solutions might support them in their job to improve patient outcomes, even though AI is unlikely to replace doctors in the near future.
Gould et al., 2019 [37]	Da Vinci Surgical System	It is well known that there are numerous medical specializations and procedures where minimally invasive surgery is superior to open surgery. These variations include shorter hospital stays, reduced discomfort, fewer hernias and wound infections, a speedier return of bowel function, and a shorter recovery period before returning to regular activities. Robotic surgical systems have allowed surgeons to use less invasive procedures more often and to abandon open surgery in some specialties, most notably gynecology and urology.
Remily et al., 2021 [38]	Impact of Robotic Assisted Surgery on Outcomes in Total Hip Arthroplasty	When comparing robotic-assisted THA to traditional techniques, there were only slight reductions in LOS and expenses. However, there was little correlation between automation and increased blood transfusions and readmissions.
Kotamarti et al., 2020 [39]	Rethinking the Need for Overnight Admission After Robotic-Assisted Laparoscopic Prostatectomy	The best surgical procedure for treating localized prostate cancer (PCa) is robotic-assisted laparoscopic prostatectomy or RAP. Less than 5% of patients have problems and readmissions, according to multi-institutional series, and the majority of patients are now released from the hospital 24 hours after surgery. A number of busy surgeons have recently shown that same-day discharge (SDD) following RALP is safe. The primary advantages encompass decreased expenses and a decreased risk of nosocomial infections and hospital blunders.
Soliman et al., 2011 [40]	Radical Hysterectomy: A Comparison of Surgical Approaches After Adoption of Robotic Surgery in Gynecologic Oncology	Patients undergoing radical hysterectomy have benefited greatly from minimally invasive surgery, including a reduction in blood loss and transfusion rates; nonetheless, operating times were noticeably longer than with open radical hysterectomy.
Boggess et al., 2008 [41]	A Comparative Study of 3 Surgical Methods for Hysterectomy With Staging for Endometrial Cancer: Robotic Assistance, Laparoscopy, Laparotomy	Women undergoing endometrial cancer staging may experience less patient morbidity when using minimally invasive endoscopic surgical methods. Surgical staging with laparoscopic assistance leads to less blood loss and quicker recovery.
Advincula et al., 2007 [42]	The Role of Robotic Surgery in Gynecology	The available data supports the viability and safety of the robotic technique in gynecologic surgery. However, experience is still in its infancy, and further studies are required to assess the effectiveness in comparison to traditional laparoscopy and to help identify which patients and applications should benefit most from robotically assisted surgery.
Arms et al., 2015 [43]	Improvement in Quality of Life After Robotic Surgery Results in Patient Satisfaction	Minimally invasive surgery has several well-documented advantages, such as reduced blood loss, a shorter hospital stay, and a quicker recovery. Although there is growing recognition for robotic surgery in gynecologic oncology, little information about the quality of life (QOL) following robotic surgery is currently accessible.
Janda et al., 2010 [44]	Quality of Life After Total Laparoscopic Hysterectomy Versus Total Abdominal Hysterectomy for Stage I Endometrial Cancer (LACE): A Randomised Trial	When treating stage I endometrial cancer, TLH is more favorable than TAH in terms of adverse event profile and quality of life improvements from baseline during early and later periods of recovery.
Kornblith et al., 2009 [45]	Quality of Life of Patients With Endometrial Cancer Undergoing Laparoscopic International Federation of Gynecology and Obstetrics Staging Compared With Laparotomy: A Gynecologic Oncology Group Study	The QoL advantage of using laparoscopy to stage patients with early endometrial cancer is somewhat supported by statistically significantly better QoL across many parameters in the laparoscopy arm at 6 weeks, even though the FACT-G did not show a MID between the two surgical groups and only modest differences were found in return to work and BI between the two groups.
Dahl et al., 2015 [46]	Effectiveness of an Intermediate Care Hospital on Readmissions, Mortality, Activities of Daily Living and Use of Health Care Services Among Hospitalized Adults Aged 60 Years and Older–a Controlled Observational Study	Shorter hospital stays were made possible by the municipality's ICH, which also maintained mortality, readmission, and post-hospitalization care demands at the same level as before.
Faria et al., 2023 [47]	Patient’s Safety and Satisfaction on Same Day Discharge After Robotic and Laparoscopic Radical Prostatectomy Versus Discharge After 24 or 48 H: A Longitudinal Randomized Prospective Study	In a subset of patients with prostate cancer, same-day release was safe, and practical, and did not seem to have an impact on patient satisfaction. The Gleason score should be taken into account by surgeons when deciding whether same-day release is appropriate.
Intuitive Surgical [48]	Annual Report	R.A.S. has been utilized increasingly frequently; according to Intuitive Surgical of Sunnyvale, California, 1.25 million procedures were performed worldwide in 2020 using the da Vinci surgical system alone.
Ma et al., 2020 [49]	Machine Learning in the Optimization of Robotics in the Operative Field	The nexus of robotics-derived "big data" and machine learning (ML) is a fast-moving field of research with the potential to improve surgical quality and safety. The ultimate purpose of these investigations has been to provide fast and meaningful surgical input intraoperatively to prevent adverse outcomes. To this end, ML models have been used to provide objective and efficient surgical assessment. The selection of surgical patients has been guided by predictive machine-learning algorithms. In conclusion, machine learning (ML) enables surgical robots to acquire autonomous procedural knowledge via expert demonstrations, trial-and-error, or a combination of these two methods.
Mithany et al., 2023 [50]	Advancements and Challenges in the Application of Artificial Intelligence in Surgical Arena: A Literature Review	With its potential to improve patient outcomes and revolutionize conventional surgical techniques, artificial intelligence has become a major player in the surgical field. The review has brought to light the noteworthy influence of artificial intelligence in a number of surgical domains, from preoperative planning to postoperative analysis.
Sandip et al., 2019 [51]	*Artificial Intelligence and the Future of Surgical Robotics *	By the end of the twenty-first century, surgical robots that are therapeutically feasible should become a reality. Artificial intelligence (AI) and surgical robotics may be used to enhance surgical capability in order to improve results and expand access to care.
Abbas et al., 2020 [52]	Financial Impact of Adapting Robotics to a Thoracic Practice in an Academic Institution	Higher CM per case is driven by high acuity procedures like thoracic surgery, provided that variable costs, particularly LOS, are maintained to a minimum. Lower CMI procedures might not yield a high enough CM to balance the fixed and variable costs. Outpatient robotic surgical cases may result in large losses because the reimbursement does not equal the out-of-pocket expenses. Hospitals should endeavor to reduce overall LOS and give priority to inpatient treatments with greater CMI when allocating robotic resources.
Korsholm et al., 2018 [53]	A Systematic Review About Costing Methodology in Robotic Surgery: Evidence for Low Quality in Most of the Studies	The studies that assessed the expenses of robotic surgery had poor methodological quality. The longest follow-up period was four months, and most investigations lacked the use of comprehensive cost data overall. Seldom were important factors like purchase, robotic platform maintenance expenses, and surgical equipment utilization disclosed. Healthcare cost studies might not offer a solid basis for decision-making if they are opaque about the cost drivers they take into account.
Soomro et al., 2020 [54]	Systematic Review of Learning Curves in Robot-Assisted Surgery	Estimates of the learning curve were quite uncertain. There was a dearth of solid evidence because of study design flaws, reporting gaps, and significant variation in the approaches taken to evaluate learning curves. There is still time to develop the best quantitative techniques for evaluating learning curves in order to guide surgical education initiatives and enhance patient outcomes.
Lawrie et al., 2022 [55]	Barriers and Enablers to the Effective Implementation of Robotic Assisted Surgery	The initial adoption, integration, and maintenance of RAS in clinical practice were hampered by a number of factors, both behavioral and organizational in nature. The impact of specific obstacles and facilitators varied according to the implementation stage. These findings will help managers and physicians make the most of the expensive technology by actively anticipating and comprehending these influences.
Jenison et al., 2012 [56]	Robotic Surgical Skills: Acquisition, Maintenance, and Degradation	It is imperative to give robotic surgeons with active curricula that aim to sustain performance during periods of inactivity, as newly trained surgeons' robotic surgical abilities deteriorate quickly. This will help to assure patient safety.
El-Hakim et al., 2007 [57]	Challenges of Robotic Surgery	The current state of robotic surgery is costly and provides only marginally better outcomes than traditional methods. The robotic system in use now is cumbersome and inadequately adaptable.
Gkegkes et al., 2017 [58]	Robotics in General Surgery: A Systematic Cost Assessment	The enforcement of robotic technology in operations of widespread surgical operations constitutes a novelty that can have an effect on each surgical remedy of several pathologies and the postoperative outcomes. The robot-assisted surgical operation has severe opportunities to conform to a cost-powerful technique, particularly in centers with a wide variety of cases, no matter the simple improved fees of acquisition and maintenance.

RAS plays a significant role in public health in various ways.

Minimally Invasive Procedure

The first laparoscopic cholecystectomy occurred in 1987, marking the beginning of minimally invasive surgery. Since then, the number of laparoscopic surgeries has increased at a rate consistent with technological advancements and surgical expertise [14]. The benefits of minimally invasive surgery are well-liked by patients, physicians, and insurance providers. There are fewer incisions, a lower chance of infection, shorter hospital stays, if any, and markedly quicker convalescence. Numerous studies have demonstrated the benefits of laparoscopic surgeries, including shorter hospital stays, a faster return to work, less pain, improved cosmesis, and improved immune function after surgery [15],16,17,18].

Precision and Accuracy

Because of their extreme precision, robots can perform intricate and delicate movements that could be challenging for a human surgeon. The precision and accuracy may result in better surgical outcomes, fewer errors, and fewer problems. Better outcomes lower the risk of the issues following surgery and the need for followup care, which benefits public health overall. Without question, robotic surgery has altered surgical practice and intervention. Nowadays, many platforms are employed with varying performance and applicability to perform various processes. Due to several technological advancements, including vibration filtration, continued improvement of wrist motion freedom, motion scalability, and improved ergonomics due to a more pleasant user interface, surgeons and the medical community reported better outcomes with this procedure than conventional laparoscopy [19].

Reduced Blood Loss and Transfusions

It has been shown that minimally invasive surgery (MIS) results in less blood loss than open surgery. It helps sustain greater serum levels of albumin and globulin, essential for immune system-based infection prevention. In addition, MIS has been linked to a decreased transfusion rate [20]. Robotic surgery techniques reduce the need for blood transfusions by often decreasing blood loss during surgeries. Because it reduces the likelihood of problems from transfusions and preserves the limited supply of donated blood, it is vital for public health.

Shorter Hospital Stays

Long-term healthcare utilization costs tend to be reduced due to more excellent proficiency with robotic surgery and a decline in Emergency room and office visits [21]. The main advantages of minimally invasive surgery for the patient are less pain medication use, quicker healing, better cosmesis, and fewer wound problems. These advantages account for the widespread use of laparoscopy globally and the standard of care that minimally invasive methods are thought to provide for several procedures, including fundoplication, adrenalectomies, cholecystectomy, and bariatric surgery [22]. The benefits of RAS include shorter clinic stays, decreased blood loss, increased periodic blood exchange, and reduced pain medication [23]. Adults and children benefit from robotic surgery due to its focused approach to the target organ or location. It minimizes operating stress, reduces postoperative pain, lessens the need for postoperative opiate use, and shortens hospital stays [24]. In addition to helping specific patients, this also lessens overpopulation in medical institutions.

Enhanced Training

Currently, simulator training aims to assist students in gaining the abilities required to carry out intricate surgical procedures before practicing on actual patients. In certain domains, such as laparoscopic and endovascular surgery training, it has been shown that surgical residents perform better in the OR while using virtual reality (VR) simulators in their current configuration [25]. Sereno et al. reported a successful experiment utilizing the RP-6 (predecessor to the RP-7) remote presence robot. They have employed two different types of mentoring: (1) the typical assistance known as "active onsite mentoring," in which the skilled surgeon offers help verbally and practically by adjusting the instruments and camera's positions as needed, and (2) "passive onsite mentoring," in which the skilled surgeon restricts their support to verbal assistance without using hands to adjust the instruments' or camera's positions (a method that is more similar to the one provided by the robot). They concluded that although remote "robotic" mentoring is considered inferior to "human" mentoring, the two groups differed in more minor ways than anticipated. A remote presence robot can't take the position of an in-person mentor, but it can be a valuable tool for telementoring minimally invasive operations [26].

Accessibility

The advantages of the robotic technique are frequently noted as its increased maneuverability and enhanced vision. This enhanced vision is particularly evident in technically challenging anatomical locations like the pelvis [27]. One possible explanation for the rising use of robotic surgery is the surgeon's preference. Compared to the laparoscopic technique, the ergonomic benefits of the robotic approach have been demonstrated to reduce both physical workload and mental stress [28]. However, this expansion has happened due to the lack of solid proof from numerous RCTs, which have yet to demonstrate a clear benefit of robotics over open or laparoscopic procedures [29,30,31]. Robotic surgery is easily accessible in big cities, which are usually the locations of teaching hospitals [27]. Systems for robotic surgery have become more widely available over time, and more medical facilities are implementing this technology. Making cutting-edge medical operations more accessible to a larger community improves public health by granting access to modern surgical procedures.

Telemedicine and Remote Surgery

Master-slave technology is the other name for telesurgery or remote surgery. Robotic surgery has significantly advanced in many big countries and has substantially impacted its field in various procedures. However, a lack of surgical expertise in rural areas could increase the travel time and treatment delays for patients who need robotic surgical management, which also includes cancer patients [32]. Robot-assisted surgical management may result in treatment delays and increased travel burdens due to the concentration of robotic surgeons in urban regions [33]. By removing geographical restrictions and cutting travel time, telesurgery enables doctors to perform surgeries from a distance, increasing surgical productivity. In addition, telesurgery improves surgical results by allowing more experienced surgeons to mentor less experienced surgeons through the operative process and possibly even by giving operating surgeons real-time guidance [34]. Telesurgery can provide surgical care to a global population, particularly in inaccessible or remote regions like spaceships and rural areas or battlefields [35].

Reduced Complications and Readmissions

Cognitive-assisted robotics, considered minimally invasive, uses miniature surgical instruments to replace extensive incisions with a series of quarter-inch incisions [36]. Compared to open surgery, laparoscopic surgery reduced pain, scarring, and length of stay, enabling doctors to execute complex surgeries with more ease in the 1980s. In several surgical subspecialties today, laparoscopy-based minimally invasive surgery has emerged as the gold standard for several routine surgical operations. It is just as successful as open surgery. It is linked to shorter lengths of time in the operating room, fewer incisions, less discomfort after surgery, and higher levels of patient satisfaction [37]. Compared to traditional approaches, RAS is related to a modest decrease in length of stay (LOS) and expenses; however, no differences in surgical complications were seen. The possibility of robotics may be seen as an increasingly relevant and economical process [38]. Most robotic surgeries generally showed reduced length of stay, blood loss, and complications [39].

Patient Satisfaction

The rapid adoption of robotic surgery in gynecology can be attributed to multiple factors. Like laparoscopy, robotic surgery has benefits over open surgery, such as reduced pain, less blood loss, shorter hospital stays, and quicker recovery times [40,41]. RAS has several advantages over traditional laparoscopy, including enhanced ergonomics, articulated instruments, three-dimensional vision, and the removal of hand tremors [42]. Because of these characteristics, robotic surgery is believed to be more widely available. Compared to traditional laparoscopy, it has a shorter learning curve, so surgeons who would otherwise rely on an open approach can now provide their patients with minimally invasive surgery [43]. Research comparing the quality of life following gynecologic laparotomy versus traditional laparoscopy for similar causes reveals that patients have superior results from less invasive surgery [44,45]. Robot-assisted laparoscopy is a minimally invasive surgery that should provide benefits comparable to traditional laparoscopy in terms of quality of life. The idea is that a patient's motivation and choice of care significantly impact whether or not they are discharged from the hospital the same day after surgery. Similarly, having a solid support system after being released from the hospital is crucial to recovery during the first few days following surgery. The use of an intermediate care hospital built in a municipality was demonstrated by reducing the LOS without increasing readmissions, admissions, mortality, activities of daily living, primary healthcare utilization, or total care days [46]. Improving acceptability and the success of early discharge may be achieved by providing initial assistance in a support home under the supervision of a technical nursing assistant. Nonetheless, it is well-known that not all nursing and support homes exist globally. Thus, early outpatient followup and easy access to the medical team through electronic communication may reduce postoperative anxiety [47].

Artificial Intelligence (AI) and RAS

One of the medical specializations that produce extremely massive datasets that AI can evaluate in-depth and thoroughly is surgery. Preoperative staging (clinical, laboratory, and imaging test results of patients), intraoperative data (based on video recordings and kinematic data), and intraoperative datasets (such as operative times, morbidity and mortality, patient outcomes, and patient-reported outcome measures; the latter were introduced during the previous 40 years to offer an evaluation of the treatment received from the patient's point of view) are among the surgical datasets on these topics. AI advancements are expected to aid digital surgical techniques, such as the master-slave manipulators used in RAS. RAS has been used more frequently; in 2020, 1.25 million procedures globally were conducted with the da Vinci surgical system alone (Intuitive Surgical, Sunnyvale, CA, United States) [48]. At this point, RASs are helping surgeons by magnifying their vision, improving their agility, and reducing tool vibrations [49]. AI has many benefits, including increased diagnostic precision through genetic data analysis, which permits early detection and individualized treatment plans. Evaluating each person's risk profile makes non-invasive screening easier and lessens the need for intrusive procedures [50]. AI and machine learning (ML) are transforming the area of robotic surgery. Robotic surgeons can support human surgeons during complex surgeries by utilizing sophisticated algorithms, which lower the possibility of problems and improve results. Surgical robots use AI, ML, and deep learning (DL) to help surgeons perform complex procedures more precisely and accurately [51].

A comparison table is shown to compare the traditional surgeries versus RASs (Table 3).

**Table 3:** Difference between robotic-assisted surgeries and traditional surgeries Table credits: Alisha Handa

	Robotic-assisted surgeries	Traditional surgeries
Invasion	Minimally invasive	Highly invasive
Precision and accuracy	Higher	Decreased
Blood loss and transfusion	Reduced	Increased
Hospital stay duration	Shorter	Longer
Recovery time	Short	Extended
Complications	Less risk	Higher risk

Challenges of RAS in public health

Like any new technology, the choice to use robotic surgery must consider financial feasibility. All healthcare professionals are under increasing pressure to offer high-quality care to more patients at a lower cost in the present healthcare climate. Using new, expensive technology that might (or not) improve patient care directly competes with this mandate [52]. There needs to be more availability for robotic surgery despite its potential. High-income countries have been at the forefront of creative advancements in robotic procedures that will help improve surgical precision. However, these ideas have yet to reach low-income nations due to a lack of financial infrastructure. Because robotic surgery requires less recovery time, it is practical in the long run, but the upfront expenditures are still very high [34]. Because robotic surgery requires less recovery time, it is functional in the long run, but the upfront prices are still very high [53]. The uptake of robotic technology has been slow despite the reported benefits of RAS over traditional minimally invasive approaches and the improved hospital experience. This is mainly due to high capital and maintenance costs and uncertainty about the potential advantages of robot-assisted methods over conventional laparoscopic procedures [54].

The disruptive process of integrating RAS services into the more extensive clinical system necessitates a significant investment in personnel training, equipment expenditures, and service alignment. The successful implementation of RAS is especially difficult since it necessitates a considerable financial investment, physical adaptation to the new technology, and a significant shift in organizational and human processes and behaviors to operate with the latest systems [55]. Specific complex and highly specialized surgeries can still be better performed using the traditional method, limiting the scope of RAS use. Regular maintenance is usually necessary for robotic systems to function at their best. The robotic system might momentarily stop working during planned maintenance intervals, which would cause delays in the surgery schedule. To reduce the impact of this downtime on patient care, it must be carefully controlled [56]. The current state of robotic surgery is costly and provides only marginally better outcomes than traditional methods. The existing robotic technology must be more versatile and more convenient [57]. Apart from the initial investment, other expenses include replacement parts, continuous upkeep, and anticipated depreciation. Salaries, overhead for administration, and the cost of non-robotic instruments are additional, less obvious, but equally significant expenses [52].

The hospital's payment for using the robot directly correlates with the kind of health insurance and the health system itself, which benefits the nations without universal health care and is harmful for those with universal health care [58]. Public health facilities already have infrastructure issues that make it challenging to integrate robotic technologies smoothly. Enhancing infrastructure to support robotic surgery could necessitate extra funds and time.

## Conclusions

RAS in public health offers a complicated and transformational landscape with many benefits and drawbacks. Several possible advantages include better patient outcomes due to increased precision, decreased invasiveness, and faster recovery times. These benefits are consistent with the main objectives of public health, which are to effectively provide diverse populations with high-quality healthcare. Navigating the difficulties of integrating RAS into public health settings is crucial. To guarantee fair and widespread adoption, it is necessary to overcome financial limitations, training needs, and discrepancies in access. Additional challenges include standardization, regulatory compliance, and infrastructure changes, which need a planned and cooperative approach. Given the potential for RAS to transform healthcare provision, efforts to overcome these obstacles are imperative. Public health institutions must actively participate in comprehensive planning, education, and resource allocation as technology advances and becomes more affordable. Careful evaluation of patient awareness, legal frameworks, and ethical issues is also necessary to build trust and guarantee the proper application of this novel strategy. In summary, despite ongoing difficulties, the benefits of RAS in public health highlight its potential to change the surgical intervention environment completely. Incorporating robotics into public health practices can improve surgical outcomes, improve patient care, and further the worldwide advancement of healthcare with careful thought, investment, and teamwork.
